# Tuberculous otitis media with postaural abscess and submandibular lymphadenopathy

**DOI:** 10.4103/0970-2113.45200

**Published:** 2009

**Authors:** Sanjeev K. Verma, Vineet Mahajan, Anand N. Srivastava

**Affiliations:** *Department of Pulmonary Medicine, Chhatrapati Shahuji Maharaj Medical University, Lucknow, UP, India*; 1 *Department of Pathology, Chhatrapati Shahuji Maharaj Medical University, Lucknow, UP, India*

**Keywords:** Submandibular lymphadenopathy, tuberculous otitis media, postaural abscess

## Abstract

We are reporting a case of right-sided tuberculous otitis media with postaural abscess and multiple submandibular lymphadenopathy which has been reported very infrequently. A high level of suspicion by the treating physician is mandatory to avoid long delay in diagnosis and increased complications in the modern chemotherapy era.

## INTRODUCTION

Tuberculosis is one of the major infectious diseases with predominant involvement of lung and lymph nodes but tuberculosis of the middle ear is uncommon. Primary tuberculosis of the ear has rarely been reported and the disease is usually secondary to infection in the lung, larynx, pharynx, and nose.^1^,^2^ Ear can become infected with *Mycobacterium tuberculosis* by the bacilli invading the eustachian tube while the infant is being fed or by hematogenous spread to the mastoid process.^3^ We describe here a case of right-sided tuberculous otitis media with postaural abscess and right submandibular lymphadenopathy in a 7-year-old boy.

## CASE HISTORY

**Figure 1:**
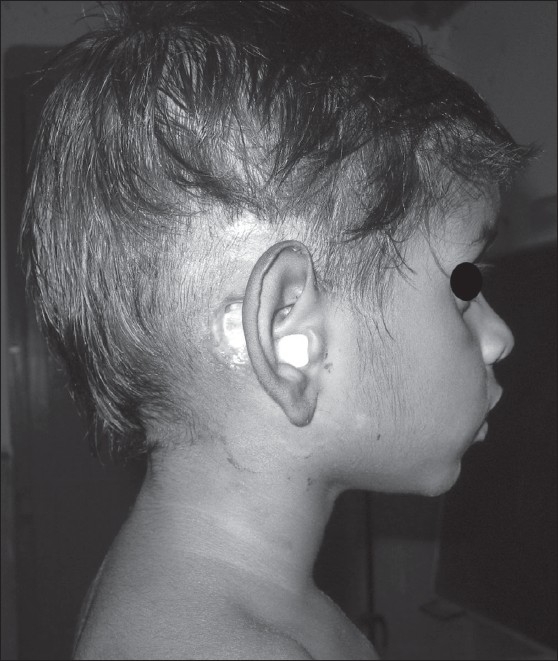
Showing ear discharge and modified radical mastoidectomy wound

A 7-year-old boy came to our department with complaints of fever and discharge from right ear of three months duration. He also had multiple right submandibular and right inguinal lymphadenopathy of two months duration. Examination of right ear showed purulent discharge and there was a fluctuant, tender swelling in postauricular area. ENT evaluation showed congested tympanic membrane with multiple perforations and there was whitish granulation tissue in the attic area. He had multiple right submandibular and right inguinal lymphnodes which were matted and nontender. Modified radical mastoidectomy (MRM) had been done in ENT department with open mastoid process to drain the pus [Figure 1].

**Figure 2:**
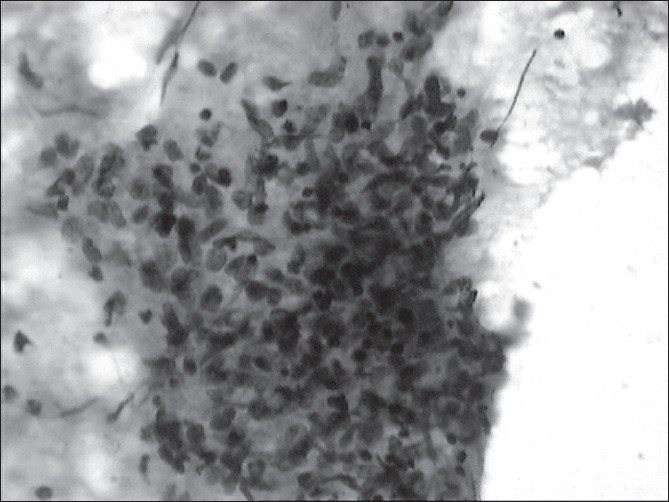
Fine nedle aspiration cytology of the enlarged submandibular lymph node showing well-formed epithelioid granulomas and few Langhan's giant cells in background of lymphocytes and histiocytes, and necrosis suggestive of tuberculous etiology

There was history of tuberculosis in the family. The patient's father was on antitubercular therapy. Laboratory investigations showed Hb, 8.2 gm/dl; TLC, 9800 /cmm; and DLC, N38 and L62. There was 18-mm induration in Mantoux test. Skiagram chest did not show any pleural or parenchymal abnormality. Specimens from ear discharge were negative for pyogenic and acid fast bacilli on smear and culture. Fine needle aspiration cytology (FNAC) of the enlarged submandibular lymph node showed well-formed epithelioid granulomas and few Langhan's giant cells in background of lymphocytes and histiocytes, and necrosis suggestive of tuberculous etiology [Figure 2].

So, the diagnosis of right-sided tuberculous otitis media with postaural abscess was made and the patient was started on three drug antitubercular therapy (Rifampicin, Isoniazid, and Pyrazinamide). After one month of ATT, patient is clinically improving and gaining weight with regression of all the lymph nodes once again confirming our diagnosis.

## DISCUSSION

Tuberculosis of middle ear is known to occur in all age groups, especially in children (50%).^4^ It is a rare condition and the exact incidence is unknown. In view of the extremely low incidence (< 1%) of ear diseases, it often precludes the diagnosis,^5^ especially in the absence of concomitant tuberculous focus elsewhere.

The route of spread of tuberculosis to middle ear has been argued for many years; the most logical route of entry of organisms being via the pharyngotympanic tube. However, hematogenous spread has also been described.^3^ Tuberculosis of the middle ear is characterized by painless otorrhea, multiple tympanic perforations, abundant granulation tissue, bone necrosis, and severe hearing loss.^6^ Demonstration of acid fast bacilli in the ear discharge is difficult. The positivity of AFB in ear discharge varies from 5–35% and on repeated examinations it improves to 50%.^7^ In our patient, it was clinical presentation, condition of tympanic membrane and especially FNAC of submandibular lymph node helped in making the diagnosis. In the past, surgery was the primary treatment to prevent neurological complications. Currently, tuberculosis of the middle ear is best treated with conservative ATT. We started our patient with ATT after getting modified radical mastoidectomy (MRM) and he has improved after one month of therapy.
